# Maternal nurturing experience affects the perception and recognition of adult and infant facial expressions

**DOI:** 10.1371/journal.pone.0205738

**Published:** 2018-10-23

**Authors:** Michiko Matsunaga, Yukari Tanaka, Masako Myowa

**Affiliations:** Graduate School of Education, Kyoto University, Kyoto, Japan; Universita degli Studi di Udine, ITALY

## Abstract

The perception and recognition of facial expressions are crucial for parenting. This study investigated whether and how maternal nurturing experience and trait anxiety influence the perception and recognition of infant and adult facial expressions. This was assessed by comparing the performance of primiparous mothers (*n* = 25) and non-mothers (*n* = 28) on an emotional face perception task. Trait anxiety was measured using the Japanese version of the State-Trait Anxiety Inventory (STAI). We found that mothers had higher recognition accuracy for facial expressions, but only of adults, not infants. Moreover, as trait anxiety increased, so did mothers’ sensitivity in perceiving facial expressions of both infants and adults. These findings suggest that maternal nurturing experience does enhance the recognition of adult emotional expressions, and an optimal level of maternal trait anxiety may enhance mothers’ sensitivity toward infants’ and adults’ emotional signals.

## Introduction

Maternal sensitivity, defined as being attuned to an infant’s physical and mental needs, is essential for positive parenting. It provides a platform to develop an effective attachment relationship to support infants’ physiological, cognitive, and social-emotional development [1–3]. Theoretically, it is crucial for mothers to effectively perceive, recognize, and respond to their infants’ cues to enhance maternal sensitivity [4]. Previous studies have shown that mothers are more responsive than are non-mothers toward infants’ sensory cues, such as crying [5] and olfactory cues [6].

Among nonverbal social cues, facial emotional expressions are also especially important for parenting. Infants have the ability to discriminate between and display facial expressions [7]. From the age of six months, infants can convey their physiological and emotional states using the six fundamental emotional facial expressions (i.e., happiness, surprise, sadness, disgust, anger, and fear) [8–9]. Moreover, infants’ faces are well-known for their unique configurational features—known as “baby schema”—that are perceived as cute and attractive [10], and this could motivate caretaking behaviors in adults [11–13]. Therefore, for mothers, perceiving and recognizing infant emotional facial expressions play an important role in appropriate and sensitive maternal attunement during the first year postpartum.

When a mother responds to her infant, she needs to first perceive the infant’s facial emotional expression as meaningful and then identify its meaning accurately. Based on an emotional information processing model, we focused on the possible strategies of both “perceptual sensitivity” and “recognition of emotional signals.” According to Adolphs [14], there are primarily two strategies of emotional processing when we perceive others’ facial expressions and determine their emotional meaning. Perceiving emotional signals enables us to detect their presence. This is a coarse but fast process for highly salient stimuli that mainly relies on the subcortical brain system. On the other hand, recognizing emotional signals enables us to understand the emotional meaning of a certain expression by identifying the emotion category (e.g., sad, happy, and angry). This precise processing needs various cognitive processes, such as memory and conceptual knowledge of emotional signals. In general, we can perceive or detect an emotional signal from a person’s facial expression, but we cannot always clearly determine the emotional category, especially when the expression is ambiguous. Regarding maternal sensitivity, mothers can sensitively perceive an infant’s cry before making an inference about the infant’s needs or the reason for crying [5]. It is also possible that mothers sensitively perceive an infant’s emotional facial expressions before accurately recognizing their emotional meaning as well. Therefore, in this study, we examined both perceptual sensitivity and recognition accuracy.

Despite both perceptual sensitivity to and recognition accuracy for infants’ emotional facial expressions being important for maternal sensitivity, the factors that influence the perception and recognition of infant emotional facial expressions remain unclear. One factor that might affect both perceptual sensitivity and recognition accuracy is mothers’ nurturing experiences. Here, nurturing experience refers to pregnancy, childbirth, and primary caregiving (i.e., breastfeeding). In general, when women become mothers, their bodies and brains experience dramatic changes. In particular, the structural and functional changes in the brain and drastic hormonal fluctuations continue from pregnancy to after childbirth. For instance, according to a neuroimaging study by Hoekzema et al. [15], pregnancy induces prolonged changes (at least for two years) in areas of a woman’s brain that are involved in recognizing other people’s emotions. Likewise, compared to non-mothers, mothers’ brain activity is higher in a broad range of areas when they are exposed to their own infants’ crying and facial stimuli [16–17]. More specifically, mothers show enhanced activity in areas associated with emotion recognition (e.g., the amygdala, temporoparietal junction, and right prefrontal cortex) and empathy (e.g., the insula). Differences between mothers and non-mothers have also been reported in relation to the hormone oxytocin. Oxytocin plays a critical role in the female reproductive system (i.e., uterine contractions and lactation) [18–19]. Moreover, oxytocin positively influences social behavior and affiliation, as it enhances the salience of social cues such as facial expressions [20–22]. Therefore, mothers’ physiological changes due to childbirth and nurturing experience may modulate their ability to perceive and recognize infants’ facial emotional expressions.

Mothers, compared to non-mothers, are more attentive to unknown infant faces [23] and more strongly evaluate the emotional valence of unknown infants’ facial expressions [24], although infant faces are generally more predisposed to capture attention than are adult faces regardless of one’s nurturing experience. Moreover, mothers can explicitly describe infants’ mental state and intentionality more than non-parents can when they observe infant behaviors (e.g., when infants play with a toy or interact with their mothers) [25–27]. These enhanced maternal abilities might be specialized in relation to infant cues. However, a recent study suggested that nurturing experience could alter perceptual sensitivity to adult facial expressions. More specifically, a longer duration of breastfeeding enhances the ability to detect and recognize happy emotional signals in adults [28]. In short, nurturing experience might enhance mothers’ perception and recognition of facial expressions not only of infants but also adults. While these separate infant and adult studies support such a possibility, to the best of our knowledge, no study has investigated this effect by examining both infants and adults simultaneously.

Another important factor that could affect mothers’ perceptual sensitivity is individual differences in anxiety. Generally, regardless of nurturing experience, anxiety is related to a robust attentional bias toward negative emotional signals such as anger, threat, and fear [29–34]. In other words, a person with higher trait anxiety is predisposed to detect negative emotional signals. These findings suggest the possibility that higher trait anxiety affects healthy mothers’ and non-mothers’ sensitivity toward negative facial expressions. Regarding the relationship between nurturing experience and anxiety, anxiety disorders affect maternal sensitivity. Mothers with generalized anxiety disorder are no less responsive to their infants’ positive cues during interaction [35]. Moreover, they perceive infants’ negative emotional signals more sensitively than do mothers without any psychiatric disorders [36–38]. To our knowledge, almost all studies on anxiety in mothers have used clinical samples. It is not clear whether mothers’ trait anxiety is also related to individual differences in their emotion perception in healthy samples. In general, anxiety is generally associated with self-related negative stimuli [34]. Therefore, mothers with higher trait anxiety might be able to detect infants’ negative expressions more sensitively than non-mothers can.

The present study aimed to identify the effect of nurturing experience and anxiety level on processing emotional signals in both infants’ and adults’ facial expressions. We used facial stimuli from infants and adults, whose intensity of happiness and sadness varied from neutral to highly intense. We measured mothers’ and non-mothers’ threshold and accuracy of detecting certain emotional signals. We directly compared both threshold and accuracy performance scores of primiparous mothers and nulliparous mothers. To understand mothers’ perceptual sensitivity (i.e., threshold scores), we also examined whether trait anxiety contributes to individual differences in such sensitivity in addition to the effect of nurturing experience. Our two main hypotheses are as follows: (a) based on prior findings of infant and adult facial expressions, we predicted that compared to non-mothers, mothers will be more sensitive and/or accurate in perceiving and recognizing facial expressions of both infants and adults; and (b) higher trait anxiety will correlate with adults’ sensitive perception of negative emotional signals (i.e., sad) in both mothers and non-mothers. Regarding infants’ sad facial expression, this correlation will be stronger in mothers than in non-mothers.

## Materials and methods

### Participants

Twenty-five primiparous mothers (mean age = 32.20 years; ranging from 24 to 43 years; *SD* = 4.07 years) and twenty-eight non-mothers (mean age = 30.18, ranging from 26 to 38 years; *SD* = 3.12 years) participated in this study. The mean age of mothers’ infants (or nurturing experience) was 8.76 months (ranging from 7 to 10 months; *SD* = 1.09 months). All mothers continued breastfeeding during the period of this study. None of the participants reported currently having any psychiatric disorders or taking any medication. One non-mother who had been a nursery teacher was excluded from the analysis to control for the variable of nurturing experience. This study was approved by the Ethics Committee of Kyoto University (No. 27-P-1) and was conducted in accordance with standards specified in the 1964 Declaration of Helsinki. All participants provided written informed consent. The participants of this study provided written informed consent to publish the details in this paper (as outlined in PLOS consent form).

### Anxiety questionnaire

Trait anxiety was assessed using the Japanese version of the State-Trait Anxiety Inventory (STAI) [39, 40]. The STAI is one of the most frequently used measures of anxiety, and it can assess two aspects of anxiety. The first is “state anxiety” (i.e., how one feels at *that moment*), which indicates the transitory nature of emotional states. This is characterized by subjective feelings of tension, apprehension, nervousness, and worry, and it temporarily intensifies in a stressful situation that is dangerous or threatening. The other is “trait anxiety” (i.e., how one *generally* feels), which refers to relatively stable individual differences in anxiety-proneness. It can assess the differences between people in their tendency to perceive a stressful situation and respond to such situations with elevations in the intensity of their state anxiety reactions. Each aspect comprises 20 items rated on a 4-point scale. A higher total score indicates a higher tendency to anxiety (note that some STAI items are reverse scored). We only used trait anxiety scores to assess individual differences in anxiety-proneness. This is because none of the participants had anxiety pathology, and the experimental situation was not threatening to the participants.

### Emotion perception task

#### Test stimuli and task

For the emotion perception task, we prepared a total of 24 facial photos (8 models × 3 facial expressions). Of the eight models, four were adults and four were infants, with each age group containing two males and two females. The three kinds of facial expressions were neutral, happy, and sad. The mean age of the adult models was 29.50 years (ranging from 25 to 33 years; *SD* = 3.42 years). We segmented images from video clips of infants used in a previous study [41]. We selected infant models whose age was around seven months to match them with that of participants’ own infants as much as possible. The mean age of the infant models was 7.05 months (ranging from 6 to 7 months; *SD* = 0.45 months).

**Fig 1:**
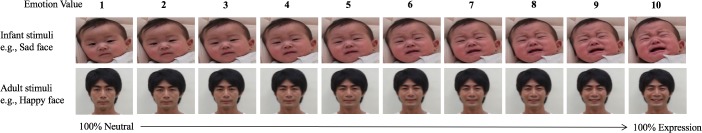
The stimuli of the emotion perception task.

The design of the emotion perception task was similar to that described in Terasawa’s study [42]. The images (400 × 266 pixels) were morphed so that the facial stimuli gradually changed from neutral to intense emotional expressions (happy or sad). This resulted in a total of 16 morphed videos (i.e., 4 adults and 4 infants × 2 happy and sad emotions). Each video consisted of 10 stages of differing emotional intensity, ranging from 100% neutral to 100% intense. We defined and labeled the stages with an emotional value of 1–10, with higher numbers indicating increased emotional intensity. For example, a 100% neutral image had the emotional value of 1 and a 100% emotional expression had the emotional value of 10 (Fig 1). FotoMorph 13.9.1, Digital Photo Software (http://www.diphso.no/FotoMorph.html) was used for this. Half of the participants were shown either male or female stimuli only.

#### Procedure

**Fig 2:**
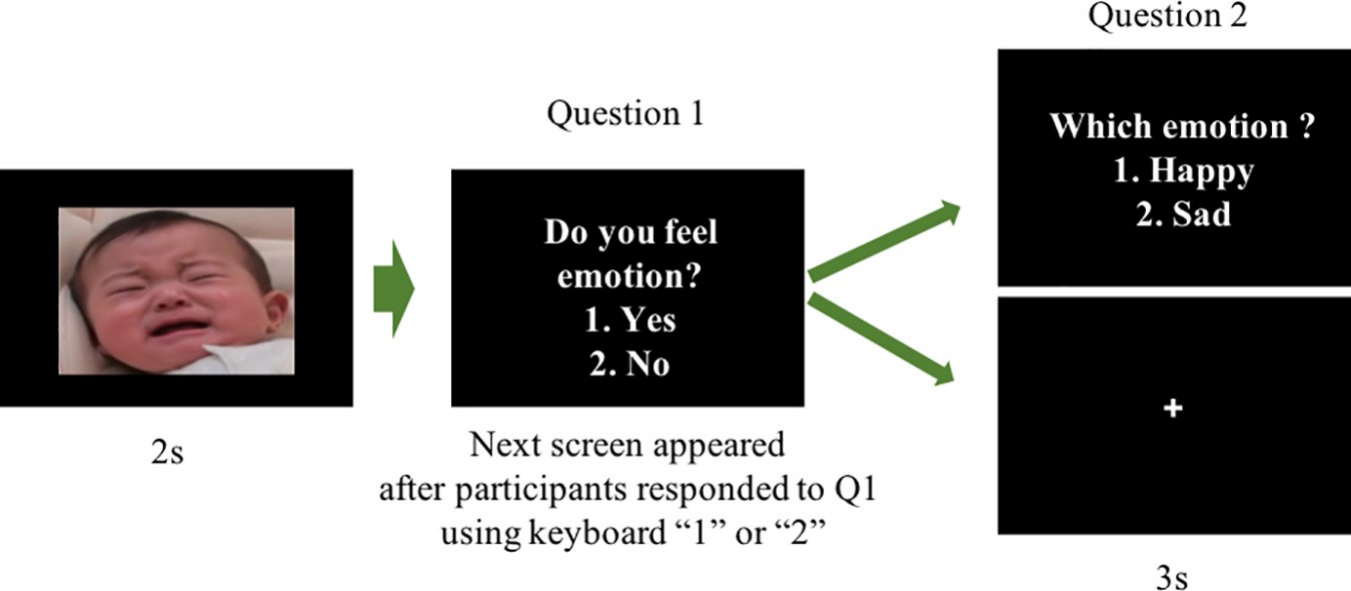
The procedure of the emotion perception task trial.

After providing informed consent, participants took the STAI questionnaire. They then participated in a sequence of tasks similar to those outlined by Terasawa [41]. For the emotion perception task, each trial was conducted as follows: (i) a stimulus was presented for 2 seconds (this stimulus could range from 1 to 10 in intensity as described above); (ii) participants responded to the question “Do you feel/think the face you are observing is expressing an emotion?” with “Yes” or “No” (Question 1) (note that we only asked the question “Do you feel an emotion?” as a prompt); (iii) if participants responded “Yes” to Question 1, a follow-up question, “Which emotion do you perceive?” was asked, which was responded to with “Happy” or “Sad” (Question 2); (iv) if participants responded “No” to Question 1, a fixation point was displayed for 4 seconds instead of the follow-up question (Fig 2). Subsequently, the next trial started. Responses were made using a numeric keypad. Each stimulus was presented five times in a random order. Thus, the total number of trials for each participant was 200 (2 models [adult and infant] × 2 emotions [happy and sad] × 10 emotional values × 5 times). After every 40 trials, participants took a short break to maintain their attention, and they could choose the length of these breaks. The gender of the models was counterbalanced between participants.

### Data preparation

#### Validity of the stimuli

To test the validity of the emotional stimuli, we conducted a pilot study based on Niedenthal’s study [43]. Twelve adults (males = 6; mean age = 22.67 years, ranging from 19 to 28 years; *SD* = 2.67 years) were asked to rate each image for levels of happiness, sadness, disgust, and anger on a 7-point scale (1 = no emotion, 7 = a high level of that emotion). The mean emotional ratings are shown in Table 1. The results of the one-way ANOVA for happy stimuli showed that all means differed from the mean happiness rating at *p* < .001. For the sad stimuli, almost all means differed from the mean sadness rating at *p* < .001. However, for adult female sad faces only, the means of the sadness and disgust ratings did not differ; yet, participants did not perceive happiness in the sad stimuli at *p* < .001. Therefore, while there may have been some confusion between sadness and disgust, the female sad faces could be sufficiently perceived as a negative emotional expression.

**Table 1:** Summary of the validity of emotional stimuli.

Infant	Male						Female					
	Neutral face		Sad face		Happy face		Neutral face		Sad face		Happy face	
	*Mean*	*SD*	*Mean*	*SD*	*Mean*	*SD*	*Mean*	*SD*	*Mean*	*SD*	*Mean*	*SD*
Happiness	1.18	0.50	1.00	0.00	6.18***	1.10	1.50	1.06	1.00	0.00	6.27***	0.88
Sadness	1.64	0.85	6.18***	0.96	1.05	0.21	2.18	1.26	5.23	1.90	1.05	0.21
Disgust	1.86	1.13	4.91	1.90	1.00	0.00	2.23	1.19	4.95	1.76	1.00	0.00
Anger	1.50	1.01	2.91	2.11	1.00	0.00	1.55	0.86	2.95	1.89	1.00	0.00
Adult	Male						Female					
	Neutral face		Sad face		Happy face		Neutral face		Sad face		Happy face	
	*Mean*	*SD*	*Mean*	*SD*	*Mean*	*SD*	*Mean*	*SD*	*Mean*	*SD*	*Mean*	*SD*
Happiness	1.14	0.47	1.36	0.73	5.77***	1.23	1.64	0.85	1.09	0.43	5.86***	1.04
Sadness	1.32	0.89	4.68***	1.29	1.14	0.64	1.50	0.74	5.27***	1.52	1.00	0.00
Disgust	1.73	0.94	2.82	1.56	1.05	0.21	1.55	0.74	3.50	1.68	1.09	0.43
Anger	1.59	0.85	1.45	0.67	1.00	0.00	1.41	0.73	2.23	1.19	1.00	0.00

*** *p* < .001

#### Analysis of sensitivity

In this study, we defined sensitivity to emotional signals as an index of perception of emotional signals, which indicates the threshold of detecting facial emotional signals. As an index of emotional sensitivity, we followed the method employed by Terasawa [42] to determine the threshold score for each emotion. Participants were presented with images ranging in emotional values from 1 to 10, and the lowest detected value was determined as the threshold score for that emotional category. In order to increase the validity, each image was presented five times, and the threshold score was calculated by the number of detection responses (i.e., “Yes” response to Question 1). Three or more detection responses were regarded as “detected,” two were regarded as “sign of detection,” and once or less were regarded as “not detected.” The lowest “detected” emotional value was determined as the threshold score, with -0.5 adjustments when there was a “sign of detection.” For example, when there was a “sign of detection” at emotional value 4 and “detected” at emotional value 3, the threshold score was determined as 3.5.

To test the effect of nurturing experience on perceptual sensitivity for emotional facial expressions, we compared mothers’ and non-mothers’ threshold scores. We performed an ANOVA for the threshold of emotional sensitivity. The between-subject factors were nurturing experience (mothers vs. non-mothers) and gender of stimuli (male or female). The within-subject factors were model category (adult and infant) and emotion category (happy and sad). Regarding the nurturing experience, the duration of nurturing experience (i.e., infant’s age) might affect mothers’ task performance and could be a potential factor. We performed Spearman’s correlation analysis as a supplementary analysis of the relationship between mothers’ sensitivity scores and infants’ age. However, there was no correlation between the two variables (Adult-happy, *r* = —.04, *p* = .83; Adult-sad, *r* = .26, *p* = .21; Infant-happy, *r* = .23, *p* = .28; Infant-sad, *r* = .17, *p* = .41). Therefore, we did not consider the factor of infant’s age in our analyses.

Second, to test the effect of individual differences in anxiety, we performed a hierarchical multiple regression analysis to examine the following two points: (1) whether trait anxiety was associated with emotional sensitivity, and (2) whether the relationship between trait anxiety and emotional sensitivity differed according to nurturing experience (mothers vs. non-mothers). The threshold score was entered as the dependent variable, and for the independent variables, we entered trait anxiety scores, nurturing experience, and their interaction. The trait anxiety variable was first centered, and nurturing experience was used as a dummy variable. The analyses were conducted in two steps. First, the main effect of trait anxiety and nurturing experience was entered at Step 1. Second, the interaction between anxiety and nurturing experience was entered at Step 2. For each step, the entered variables were considered valid if the *F* values for Δ*R*^*2*^ were significant. We verified the availability of the interaction terms by performing an ANOVA with the coefficient of determinations. If there was a significant effect of the interaction terms, we performed a simple slope analysis. This analysis was performed for each type of stimuli.

#### Analysis of accuracy (multinomial processing tree modeling)

**Fig 3:**
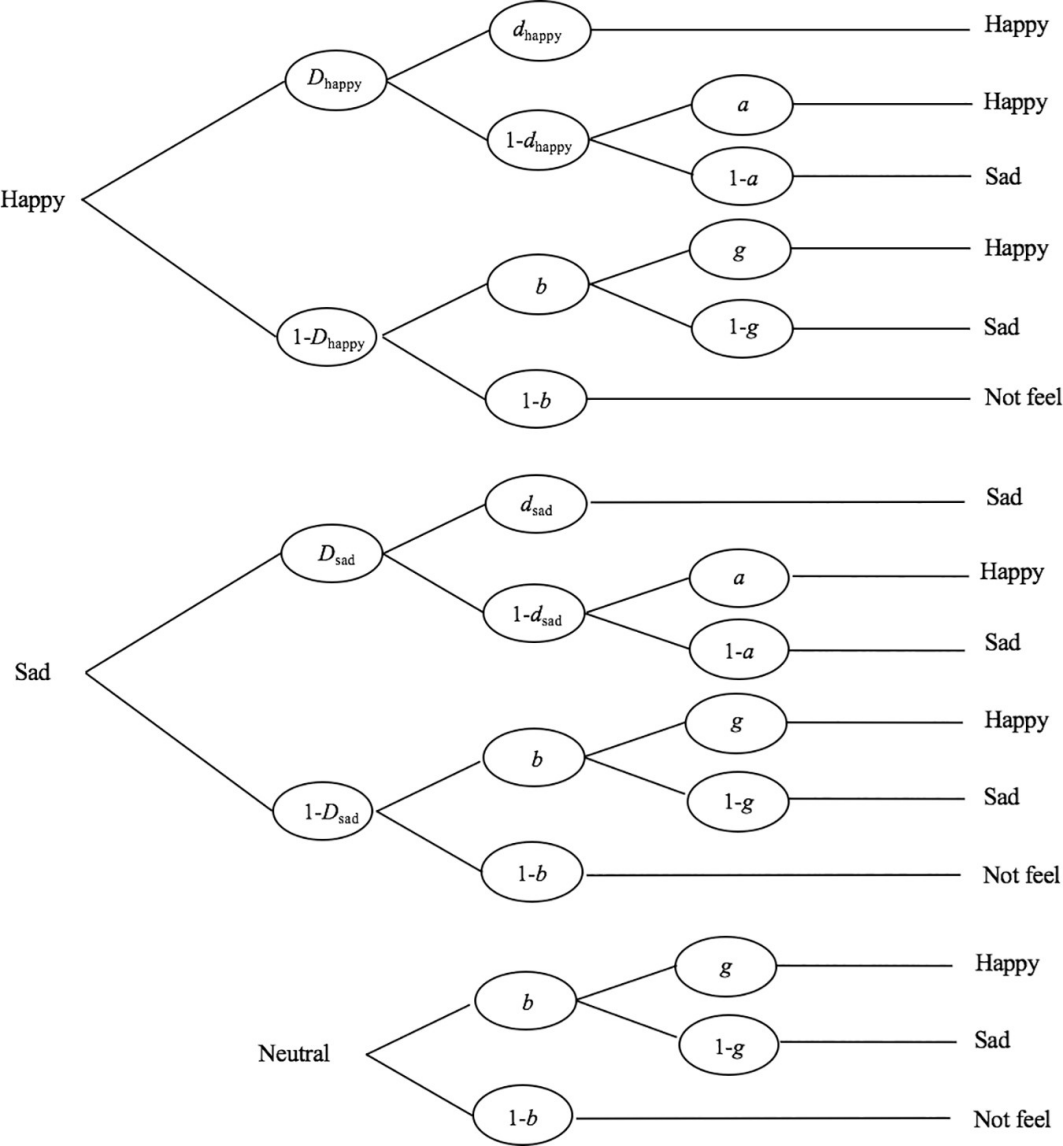
Model tree for multinomial processing tree modeling analysis. The multinomial processing tree model shows multiple cognitive pathways underlying participants’ responses to happy, sad, and neutral stimuli. *D* is the probability that participants clearly perceive and detect an emotional signal; *d* is the probability of identifying the emotional category; *a* is the probability that participants guess the emotional category when they cannot identify the emotional category (1-*d*); *b* is the probability of guessing the presence of an emotional signal when participants cannot detect an emotional signal (1-*D*); *g* is the probability of guessing the emotional category after guessing emotional signals with *b*.

We defined the accuracy of emotional signals as an index of the recognition of emotional signals, which indicates the accuracy of identifying the categories of emotional signals (e.g., happy and sad). To test the accuracy of emotion recognition, we performed a multinomial processing tree modeling analysis [44–46]. In our recent study, the emotion perception task consisted of two steps of responses: Step 1 included the detection of emotional signals, and Step 2 included identification of the emotional category. The accuracy that we aimed to evaluate was only for the responses in which participants detected the emotional signals (Step 1) and identified the emotional category (Step 2). To determine a proper index of accuracy, we removed the influence of responses in which participants failed to detect emotions at Step 1. The multinomial processing tree modeling is a good method for dealing with such data. It can evaluate and compare the accuracy by separating the different processes of responses. We used the same model as that of Dodson et al.’s study [46] (Fig 3) because their task protocol was the same as ours (for more precise information about the model, refer to [45–48]). To examine whether the accuracy of emotional identification differs according to nurturing experience, we first tested the goodness of fit for the mothers’ model and non-mothers’ model with our data. Second, we examined whether the parameter *d* (which predicts the probabilities of proper identification of emotional signals) was different between mothers and non-mothers. Infant faces and adult faces were separately examined. All hypotheses were tested using the *G*^*2*^ statistic [44]. *G*^*2*^ represents the degree of “misfit” of a model to the data, and it increases when the degree of discrepancy between the observed and predicted probabilities is bigger, following a χ^2^ distribution. The fit of the model (Fig 3) to the data was deemed satisfactory if *G*^*2*^ did not differ significantly from 0 according to a χ^2^ test. The multinomial processing tree modeling analysis was performed using multiTree (version 0.46) [49].

## Results

### Emotional sensitivity

**Fig 4:**
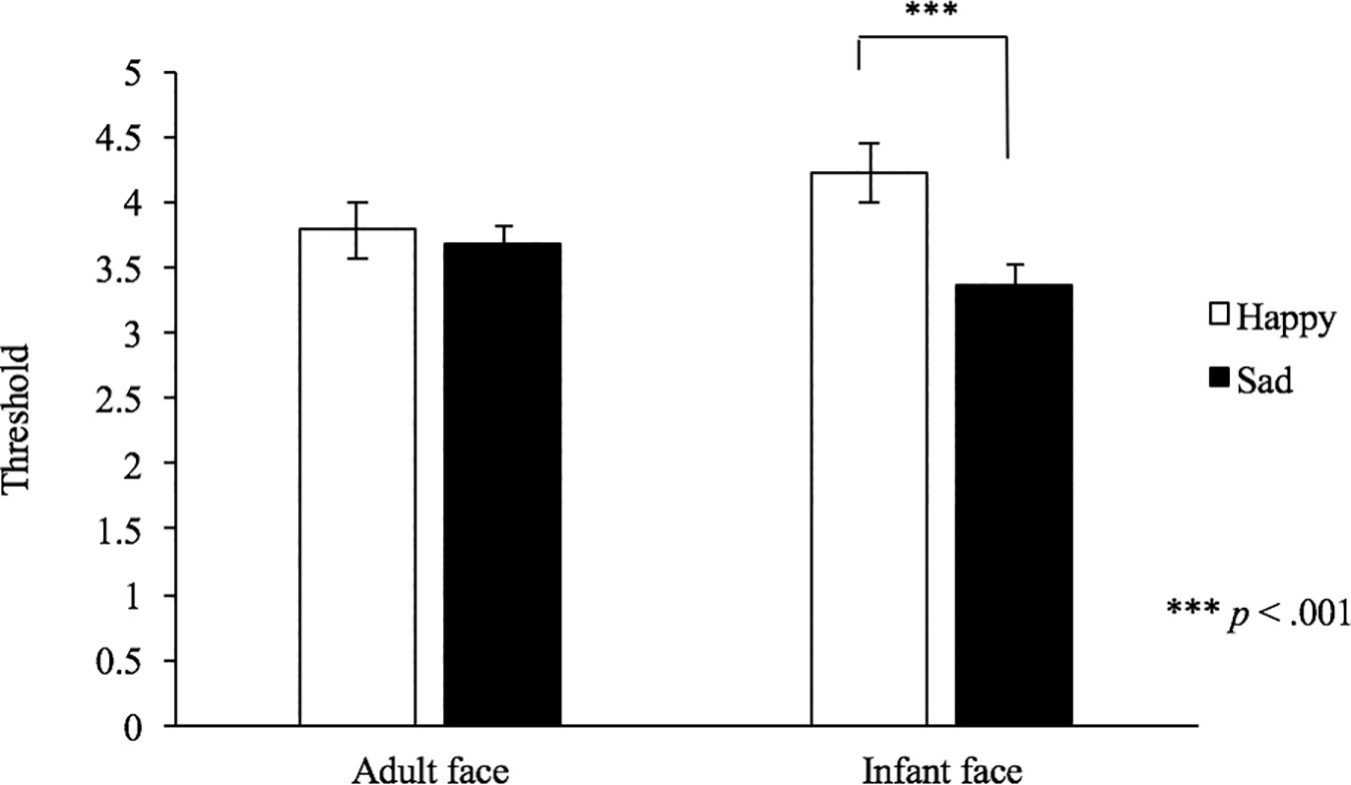
Difference in perceptual sensitivity for infant and adult facial expressions. The vertical axis indicates the detection thresholds.

Table 2 shows the mean threshold scores. We found no significant difference in nurturing experience (mothers vs. non-mothers) (*F*_1, 49_ = .08, *p* = .78, η^2^ = .00) and gender of the stimuli (male or female) (*F*_1, 49_ = .99, *p* = .33, η^2^ = .02). There was a significant main effect of emotion category (*F*_1, 49_ = 16.50, *p* < .001, η^2^ = .25), along with an interaction between the model and the emotion category (*F*_1, 49_ = 8.77, *p* = .01, η^2^ = .15). A simple main effects analysis indicated that the thresholds for infant-sad stimuli were significantly lower than those for infant-happy stimuli (*F*_1,49_ = 34.33, *p* < .001, η^2^ = .41) (Fig 4), although the thresholds of emotional stimuli were not significantly different between mothers and non-mothers.

**Table 2:** Summary of trait anxiety scores and threshold.

	Mother		Non-mother	
	*Mean*	*SD*	*Mean*	*SD*
**STAI**				
Trait Anxiety	42.16	8.07	41.89	8.24
**Threshold**				
Adult Happy	3.94	1.49	3.80	1.34
Adult Sad	3.72	0.74	3.63	1.10
Infant Happy	3.92	1.65	4.48	1.99
Infant Sad	3.34	1.21	3.39	1.29

Regarding the trait anxiety scores, the t-test showed no significant difference between mothers and non-mothers (*t*(51) = .12, *p* = .91, *d* = .02) (Table 2).

We performed a hierarchical multiple regression analysis to investigate the effect of trait anxiety and nurturing experience on sensitivity to emotional facial expression. The overall results showed no significant main effect of the nurturing experience after controlling for trait anxiety scores based on the results of Step 1 (Table 3).

**Table 3:** Summary of hierarchical multiple regression analysis.

	Adult-Happy TH			Adult-Sad TH			Infant-Happy TH			Infant-Sad TH		
	*β*	*SE*	*t*	*β*	*SE*	*t*	*β*	*SE*	*t*	*β*	*SE*	*t*
Step1												
trait anxiety	-0.05	0.02	-0.62	-0.06	0.02	-2.50*	0.16	0.03	0.63	0.02	0.02	-0.10
nurturing experience	-0.09	0.39	-0.36	-0.33	0.25	-0.42	0.09	0.51	1.12	-0.01	0.35	0.15
	Δ*R*^*2*^ = 0.01, Δ*F*_*2*,*50*_ = 0.25			Δ*R*^*2*^ = 0.11, Δ*F*_*2*,*50*_ = 3.20*			Δ*R*^*2*^ = 0.03, Δ*F*_*2*,*50*_ = 0.81			Δ*R*^*2*^ = 0.00, Δ*F*_*2*,*50*_ = 0.02		
Step2												
trait anxiety	-0.05	0.03	-2.16*	-0.06	0.02	-1.70	0.15	0.05	-0.75	0.02	0.03	-2.10*
nurturing experience	-0.09	0.37	-0.38	-0.33	0.25	-0.42	0.08	0.50	1.13	-0.02	0.33	0.15
trait anxiety × nurturing experience	0.32	0.05	2.34*	0.00	0.03	0.03	0.22	0.06	1.61	0.37	0.04	2.75**
	Δ*R*^*2*^ = 0.10, Δ*F*_*3*,*49*_ = 2.01			Δ*R*^*2*^ = 0.00, Δ*F*_*3*,*49*_ = 2.09			Δ*R*^*2*^ = 0.05, Δ*F*_*3*,*49*_ = 1.42			Δ*R*^*2*^ = 0.13, Δ*F*_*3*,*49*_ = 2.54		
	*R*^*2*^ = 0.11			*R*^*2*^ = 0.11			*R*^*2*^ = 0.08			*R*^*2*^ = 0.13		
	*F*_*1*,*49*_ = 5.48*			*F*_*1*,*49*_ = 0.00			*F*_*1*,*49*_ = 2.60			*F*_*1*,*49*_ = 7.57**		

*β*, standardized regression coefficient; Δ*R*^*2*^, change value of *R* square; Δ*F*, change value of *F*-value. TH is an abbreviation for threshold.

* *p* < .05

** *p* < .01.

**Fig 5:**
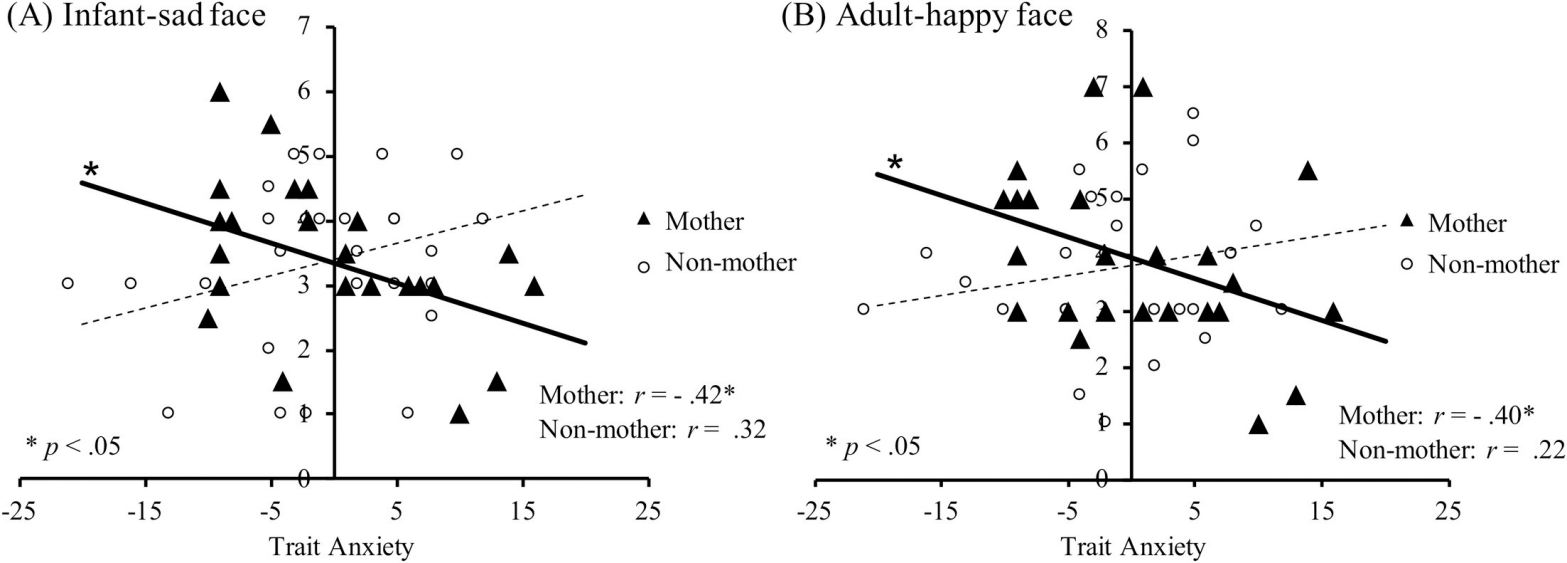
Relational differences between perceptual sensitivity and anxiety in mothers and non-mothers. Scatter plots and regression lines showing the relationship between trait anxiety scores and (A) detection thresholds for infant-sad faces (*r* = -0.42, *p* < .05); (B) detection thresholds for adult-happy faces (*r* = -0.40, *p* < .05). The horizontal axis indicates trait anxiety scores centering at the grand mean. The vertical axis indicates the detection thresholds; black triangles indicate the mothers’ group (*n* = 25), and white circles indicate the non-mothers’ group (*n* = 28).

Regarding sensitivity to adult-sad stimuli, only the first step significantly predicted the threshold scores (Δ*R*^*2*^ = 0.11, Δ*F*_2,50_ = 3.20, *p* = .04). There was a negative linear relationship between trait anxiety scores and threshold scores for adult-sad stimuli regardless of the nurturing experience. However, for sensitivity to infant-sad stimuli, the Δ*R*^*2*^ variation between Steps 1 and 2 was significantly different. Furthermore, Δ*R*^*2*^ significantly increased in Step 2 (Δ*F*_1,49_ = 7.57, *p* = .01). The main effect of trait anxiety scores was significant (*t*(49) = -2.10, *p* = .04), suggesting that there was a negative relationship between trait anxiety scores and sensitivity to infant-sad stimuli. The interaction effect between trait anxiety scores and nurturing experience was also significant. The simple main effect indicated that there was a negative linear relationship between trait anxiety scores and thresholds only in mothers (simple slopes = -0.06, *t*(49) = -2.10, *p* = .04) (Fig 5A).

Regarding sensitivity to adult-happy stimuli, the Δ*R*^*2*^ variation between Steps 1 and 2 was significantly different. Therefore, Δ*R*^*2*^ significantly increased in Step 2 (Δ*F*_1,49_ = 5.48, *p* = .02). The main effect of trait anxiety scores was significant at Step 2 (*t*(49) = -2.16, *p* = .04), suggesting that there was a negative effect of trait anxiety scores on sensitivity to adult-happy faces. The interaction effect between trait anxiety scores and nurturing experience was also significant. A simple main effects analysis indicated that there was a negative linear relationship between trait anxiety scores and emotion detection thresholds only in mothers (simple slopes = -0.07, *t*(49) = -2.16, *p* = .04; Fig 5B). Regarding sensitivity to infant-happy stimuli, there was no relationship between detection threshold scores, trait anxiety, and nurturing experience.

### Accuracy of emotional recognition (multinomial processing tree modeling)

**Fig 6:**
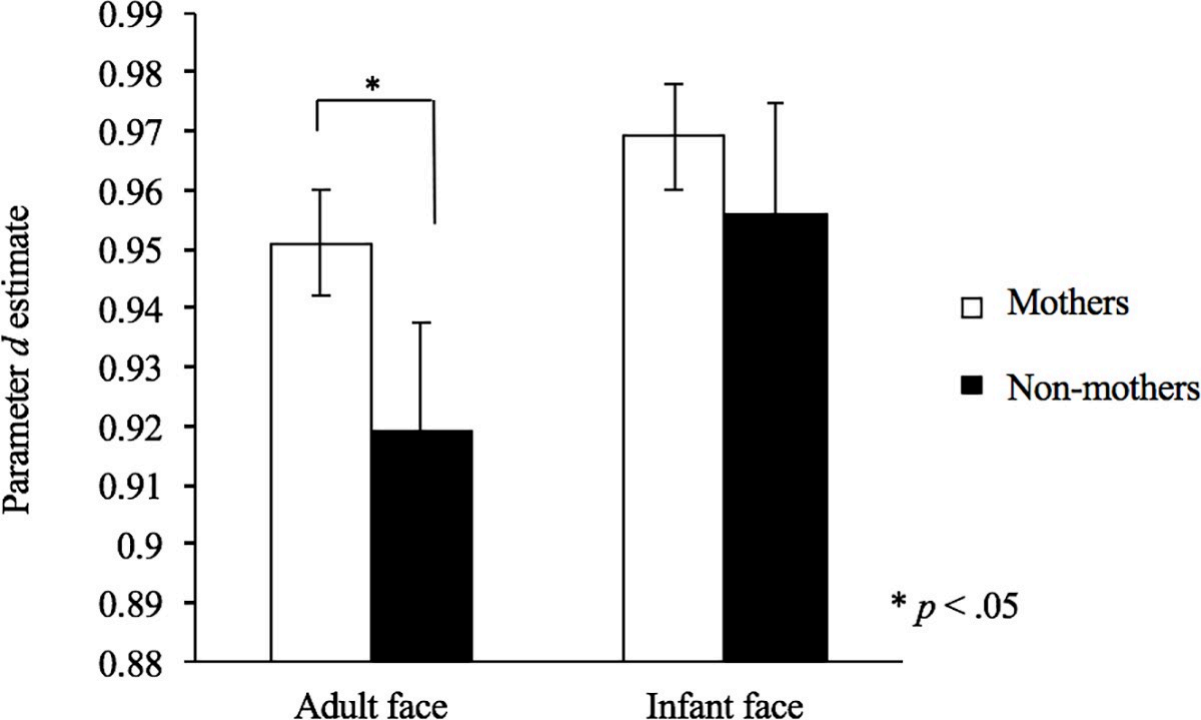
Difference in perceptual accuracy between mothers and non-mothers by multinomial processing tree modeling. The vertical axis indicates the accuracy of emotion recognition, which is represented by parameter *d* estimate. Mothers showed higher accuracy for recognizing adult emotional stimuli than did non-mothers.

Table 4 shows the observed probabilities for detecting each category of emotion. The fit of the model to the data was satisfactory for adult facial stimuli (*G*^*2*^(4) = 15.74, *p* < .01) and infant facial stimuli (*G*^*2*^(4) = 58.23, *p* < .01). We examined the effects of nurturing experience (mothers or non-mothers) on the *d* parameter estimates using the χ^2^ test (similar to Dodson et al.’s study [46]). Regarding the adult faces, we found significant differences between mothers and non-mothers in parameter *d* (*G*^*2*^(1) = 4.03, *p* < .05). These findings suggest that mothers showed higher accuracy for recognizing adult emotional stimuli than did non-mothers (mothers: *d* = .95, *SD* = .01; non-mothers: *d* = .92, *SD* = .01). For infant facial stimuli, no significant difference was found between the mothers and non-mothers in parameter *d* (*G*^*2*^(1) = .42, *p* > .05) (Fig 6), suggesting that there was no effect of nurturing experience on the accuracy of recognizing infant emotional facial stimuli.

**Table 4:** Observed probabilities for detecting each category of emotional response.

Face	Emotion	Response	Mother	Non-mother
Adult	Happy	Happy	**0.67 (0.14)**	**0.73 (0.14)**
		Sad	0.07 (0.12)	0.04 (0.08)
		Neutral	0.26 (0.13)	0.23 (0.13)
	Sad	Happy	0.01 (0.01)	0.01 (0.02)
		Sad	**0.77 (0.07)**	**0.75 (0.10)**
		Neutral	0.22 (0.07)	0.24 (0.10)
	Neutral	Happy	0.02 (0.04)	0.04 (0.14)
		Sad	0.05 (0.12)	0.03 (0.07)
		Neutral	**0.94 (0.13)**	**0.93 (0.19)**
Infant	Happy	Happy	**0.68 (0.13)**	**0.66 (0.15)**
		Sad	0.05 (0.08)	0.04 (0.07)
		Neutral	0.27 (0.14)	0.30 (0.16)
	Sad	Happy	0.02 (0.06)	0.03 (0.04)
		Sad	**0.78 (0.12)**	**0.79 (0.10)**
		Neutral	0.20 (0.11)	0.19 (0.11)
	Neutral	Happy	0.04 (0.10)	0.05 (0.09)
		Sad	0.11 (0.16)	0.11 (0.22)
		Neutral	**0.85 (0.18)**	**0.85 (0.26)**

Boldface values indicate the observed probabilities for correct responses. Values in parentheses indicate standard deviations.

## Discussion

The present study investigated whether nurturing experience affects perceptual sensitivity to and recognition accuracy of facial expressions, as well as individual differences with respect to the effect of trait anxiety on perceptual sensitivity. Regarding perceptual sensitivity to facial expressions, the results partially support our hypothesis. Although there was no main effect of nurturing experience, we found positive correlations between trait anxiety and perceptual emotional sensitivity for infant-sad and adult-happy faces only in mothers. The findings regarding infant-sad facial expressions are consistent with our hypothesis that mothers with higher trait anxiety are more sensitive in perceiving infant negative emotional signals. These results can be explained as follows. An infant’s face is characterized as inducing high arousal levels and attracting more attention compared to adult faces, and mothers showed heightened attention toward infant faces compared to did non-mothers [23, 50]. In addition, high anxiety mothers are inclined to perceive infant emotional facial expressions more negatively than low anxiety mothers are [51]. Altogether, our results could suggest that mothers with higher trait anxiety might be predisposed to attend to and perceive an infant’s negative emotion signals daily, regardless of whether the infant is theirs or not. Importantly, higher trait anxiety reported in the present study is on the lower end of the spectrum according to the general criteria. Although excessive sensitivity to negative stimuli could lead to severe stress [52], healthy levels of higher trait anxiety might enable mothers to display higher sensitivity to their infants. Interestingly, at least one animal study has reported that rat mothers with higher trait anxiety show dedicated maternal care [53].

Regarding adult positive facial expressions, we found that mothers with higher trait anxiety were more sensitive to perceiving adult happy faces. This was surprising because trait anxiety is generally related only to negative stimuli. To explain the reason behind this, several previous studies seem to provide valuable clues. For example, breastfeeding experience and oxytocin also affect mothers’ anxiety [54–56]. In addition, accumulated breastfeeding experience and drastic hormonal changes (e.g., oxytocin) might enhance mothers’ sensitivity to positive emotional signals [26, 57]. In general, breastfeeding enhances mothers’ oxytocin levels [19], and all participants in our study were breastfeeding mothers. Therefore, it is possible that mothers’ breastfeeding experience and enhanced oxytocin levels could affect the relationship between anxiety and sensitivity to positive emotion signals. However, further research is needed to examine the relationships among anxiety, sensitivity to positive stimuli, and hormonal changes in mothers [55]. It is also necessary to clarify how and what nurturing behaviors (e.g., breastfeeding) influence mothers’ physiological and psychological changes.

Concerning other facial expressions, we also found a negative correlation between anxiety and perceiving adult sad emotions, regardless of nurturing experience. As hypothesized, women with higher trait anxiety were more sensitive in perceiving adult negative emotional signals. Our results confirmed the negative bias of anxiety to negative stimuli as reported in previous research. Sensitivity to adult negative emotion signals was modulated by individual differences involving trait anxiety [30]. On the other hand, concerning infant-happy faces, there was no correlation between anxiety and emotional sensitivity. This is also consistent with the findings of previous studies that maternal anxiety symptoms did not influence the recognition of infant-happy emotional signals [51]. It makes sense that there was no relationship between anxiety and infant positive facial expressions. This is because anxiety is generally associated with self-related negative stimuli [34], and infant-happy emotions enhance the baby schema effect that makes adults perceive infants as “cute” [58], and therefore, both mothers and non-mothers are motivated to look longer at infant faces [59, 60].

However, as stated above, we found no main effect of nurturing experience in relation to unknown infant facial expressions, with respect to sensitivity or accuracy. This finding is inconsistent with studies that reported that mothers can perceive the valence of infants’ facial expression more sensitively than non-mothers can [24]. To explain this inconsistent finding, one possibility is that nurturing experience may only affect mothers when looking at their own infants. For instance, Montirosso et al. [61] reported that mothers with higher maternal sensitivity scores showed greater sensitivity only to their own infants’ body configurations. Neuroimaging studies have also shown that mothers’ brain activity differs depending on whether the stimuli are of their own infant or not [16, 62, 63]. These findings, therefore, suggest that the effect of maternal nurturing experience may be limited to the facial expressions of one’s own infant. Keeping in mind the baby schema effect [59, 60, 64], and that women are generally better at processing emotional stimuli than men are [65–67], it is perhaps unsurprising that both mothers and non-mothers could perceive and interpret emotional expressions of unknown infants.

Another possibility worth considering is that the nurturing experience effect may only be observed when mothers consider infants’ emotional states using not only facial expressions but also contextual clues (e.g., the infant’s voice, the place where the infant is, and what the infant is doing). This possibility is supported by previous studies that showed that mothers were more likely than non-mothers to recognize an infant’s mental state when they freely described their thoughts while watching a short movie on infants’ behaviors in a daily life situation [27, 68]. However, in this study, we asked participants to choose only one emotional category from a still facial image. Hence, it could be suggested that non-mothers can perceive and interpret infant emotional expressions like mothers do when they judge emotion categories only from facial expressions.

On the other hand, we found that nurturing experience enhanced recognition accuracy for adult facial expressions. This is consistent with the results of Pearson et al.’s study [69]. They found that the accuracy of identifying adult facial expressions increased during the perinatal period. In addition, our findings are also consistent with those of Krol et al.’s study [28]. They found that long-lasting breastfeeding experience increased mothers’ ability to identify emotional signals of adults sensitively (note that our participants were also mothers who breastfed every day). In daily life, infants’ emotional signals through facial expressions are ambiguous and difficult to understand [70]. Therefore, cognitive reasoning ability is necessary to perform adequate nurturing behaviors [71]. Furthermore, gestational alteration in neural activity and brain structures could enhance mothers’ ability to recognize others’ facial expressions [15]. Why, then, was mothers’ higher recognition of emotional facial expressions found only for adult facial expressions but not for infant ones? One possible reason is that we asked the participants to detect and infer others’ emotions at a conscious level. As for infant facial expressions, nurturing effects may appear as unconscious responses that primarily rely on the subcortical and limbic brain system (e.g., the amygdala) [17]. Therefore, mothers’ higher recognition of emotional facial expressions may be reflected in their enhanced recognition accuracy for adult facial expressions but not for infant ones.

In addition to the limitations discussed above, given that we used unfamiliar infant faces rather than mothers’ own infants, we could not examine the effect of accumulated experience with children (e.g., nursery school teachers, multiparous mothers, and aunts). Our participants were primiparous mothers, and their experience of looking at infant faces was less than 10 months. There is a possibility that their amount of experience was not enough to induce a nurturing effect on their perception and recognition of unknown infants’ facial expressions. Likewise, it is also unclear whether non-mothers’ accumulated experience with infants enhances their emotion recognition of infant faces. Future studies should identify the effect of the following two factors: biological changes specific to mothers, and accumulated experience of daily interactions with infants.

## Conclusion

In conclusion, the present study investigated whether nurturing experience and trait anxiety influence one’s recognition of infant and adult facial expressions. We found a negative relationship between anxiety and perceptual sensitivity to infant-sad and adult-happy emotions only in mothers. We also found that nurturing experience enhances recognition accuracy for adult facial expressions. These findings suggest that nurturing experience affects mothers’ ability to recognize adults’ emotional cues, and optimal levels of trait anxiety modulate their perceptual sensitivity to both infants’ and adults’ emotional cues.
